# Performance of dispatcher-assisted cardiopulmonary resuscitation integrating with mouth-and-nose covering instructions during the COVID-19 pandemic: a population-based retrospective study

**DOI:** 10.1186/s12873-025-01296-8

**Published:** 2025-07-26

**Authors:** Hideki Asai, Hidetada Fukushima, Yasuyuki Kawai, Keita Miyazaki, Kouji Yamamoto, Arisa Kinoshita, Hirozumi Okuda

**Affiliations:** Department of Emergency and Critical Care Medicine, Nara Medical University, Shijo-cho, 840, Kashihara City, Nara 634-8522 Japan

**Keywords:** Out-of-hospital cardiac arrest, Aerosol-borne infection, Pandemics, Infection prevention, Emergency medical dispatch

## Abstract

**Background:**

The coronavirus disease 2019 (COVID-19) pandemic, which emerged in late 2019, compelled people to change their behavior globally. Due to concerns about potential aerosol transmission during chest compressions, a modified dispatcher-assisted cardiopulmonary resuscitation (DACPR) protocol incorporating mouth-and-nose covering instructions was introduced in Nara, Japan. This study examined its impact on DACPR performance during the COVID-19 pandemic.

**Methods:**

This is a retrospective before-after study. DACPR performance data from the Nara Wide Area Fire Department were analyzed, comparing the non-pandemic period (March 2019 to February 2020) with the pandemic period (November 2020 to October 2021). The primary outcome was the time from emergency call acceptance to the first chest compression (T3). Secondary outcomes included the time to cardiac arrest recognition (T1), the time to start of DACPR instructions (T2), DACPR implementation rate, and adherence to infection prevention instructions.

**Results:**

The implementation of the modified protocol did not significantly alter the overall DACPR rate (406, 50.3% in the non-pandemic vs. 390, 47.2% in the pandemic; *p* =.214). Although the difference was relatively small, a statistically significant prolongation of T3 was observed during the pandemic period (246.0 s vs. 261.5 s, *p* <.05). Compliance with mouth-and-nose covering instructions among dispatchers was relatively low (43.1%). Among cases where such instructions were provided, only 21.4% of bystanders fully adhered to the protocol (both the bystander and the patient covering their mouth and nose). However, dispatcher-provided instructions significantly increased the likelihood of bystanders wearing masks and covering the patient’s mouth and nose. Multivariable analysis did not identify the protocol implementation as a significant factor influencing T3.

**Conclusions:**

This study demonstrated that the modified DACPR protocol incorporating infection prevention measures was associated with a statistically significant delay of approximately 15.0 s in CPR initiation. However, given the low adherence rate, the overall impact of these measures on DACPR performance was limited. These findings highlight the need to increase adherence to infection prevention measures while minimizing delays in life-saving interventions, particularly during pandemics caused by airborne pathogens.

## Background

Bystander cardiopulmonary resuscitation (BCPR) can improve survival rates and favorable neurological outcomes in sudden out-of-hospital cardiac arrest (OHCA) [1–3]. Dispatcher-Assisted Cardiopulmonary Resuscitation (DACPR), or BCPR with dispatch assistance over the phone, contributes significantly to BCPR before emergency medical service (EMS) arrival and has contributed to improving survival and favorable neurological outcomes in various communities [4–8].

The COVID-19 pandemic triggered widespread behavioral changes, including a hesitancy to perform CPR due to infection risk. This has been linked to reduced BCPR rates and lower survival in OHCA cases [9–13]. Considering the concerns about the potential for aerosol transmission during chest compressions [14, 15], the European Resuscitation Council and others have recommended covering the patient’s nose and mouth with a mask, handkerchief, or towel before starting chest compressions, and bystanders are also advised to wear masks to minimize the risk of aerosol transmission [16]. Under these recommendations, we changed the DACPR protocol in Nara, Japan, in May 2020. This modified protocol advises callers to cover the mouth and nose of patients with OHCA with a cloth or equivalent material and for bystanders to wear masks prior to chest compressions. Although the modified DACPR protocol is consistent with the above recommendations, it may delay immediate BCPR. This delay cannot be overlooked, as the outcomes of patients with OHCA depend on prompt BCPR. However, no studies have evaluated whether the DACPR protocol integrated with infection prevention can delay DACPR initiation.

This study examined how the modified DACPR protocol, requiring covering the patient’s mouth and nose before chest compressions, affected the time to initiate bystander chest compressions and bystanders’ compliance with infection prevention measures in Nara, Japan.

## Methods

### Study design

We conducted a retrospective, population-based observational study to evaluate changes in DACPR performance before and during the COVID-19 pandemic. Two time periods were compared: the pre-COVID-19 period (March 2019 to February 2020) and the COVID-19 period (November 2020 to October 2021). This before-and-after design was used to assess the impact of a modified DACPR protocol introduced during the pandemic.

DACPR was provided based on a standardized dispatcher protocol in Nara Prefecture, which included stepwise verbal instructions for chest compressions following recognition of cardiac arrest. Detailed protocol and measurement definitions are described in the Data Sources and Measurements section.

### Setting

This study was conducted in the jurisdiction of the Nara Wide Area Fire Department in Nara Prefecture, Japan. The department covers approximately 870,000 residents across 18 municipalities, including urban areas such as Kashihara and Yamatokoriyama, as well as suburban and rural communities. The total geographic area spans 3,361 km².

During the COVID-19 pandemic, a modified DACPR protocol was officially introduced in May 2020; however, the period from May to October 2020 was considered a transitional implementation phase. During this time, the number of severe COVID-19 cases in Nara Prefecture remained low, and adherence to the modified protocol was limited. As the third wave of infections began in late October, significant changes in public behavior and emergency medical practices were observed. Thus, the 1-year period from November 2020 to October 2021 was defined as the post-intervention period. During this time, Nara Prefecture experienced multiple waves of COVID-19 infections. According to official reports from Nara Prefecture (https://www.pref.nara.jp/item/299292.htm), the third wave lasted from October 26, 2020, to February 28, 2021; the fourth wave from March 1 to July 11, 2021; and the fifth wave from July 12 to December 26, 2021. The fifth wave, driven by the Delta variant, led to a substantial surge in infections, particularly in August 2021, when approximately 4,000 new cases were reported in a single month. Other surges occurred in January (1,500 cases) and May 2021 (1,500 cases), placing significant strain on the healthcare and emergency response systems. Given the substantial burden on healthcare services, particularly during the Delta-variant surge in mid-2021, this period is appropriately classified as a severe pandemic phase in the regional context. For comparison, the 1-year period from March 2019 to February 2020 was used as the control group, as the World Health Organization declared COVID-19 a pandemic on March 11, 2020.

### Study population

We included adult OHCA cases for which DACPR was attempted between January 2019 and December 2021 in the jurisdiction of the Nara Wide Area Fire Department.

**Fig. 1:**
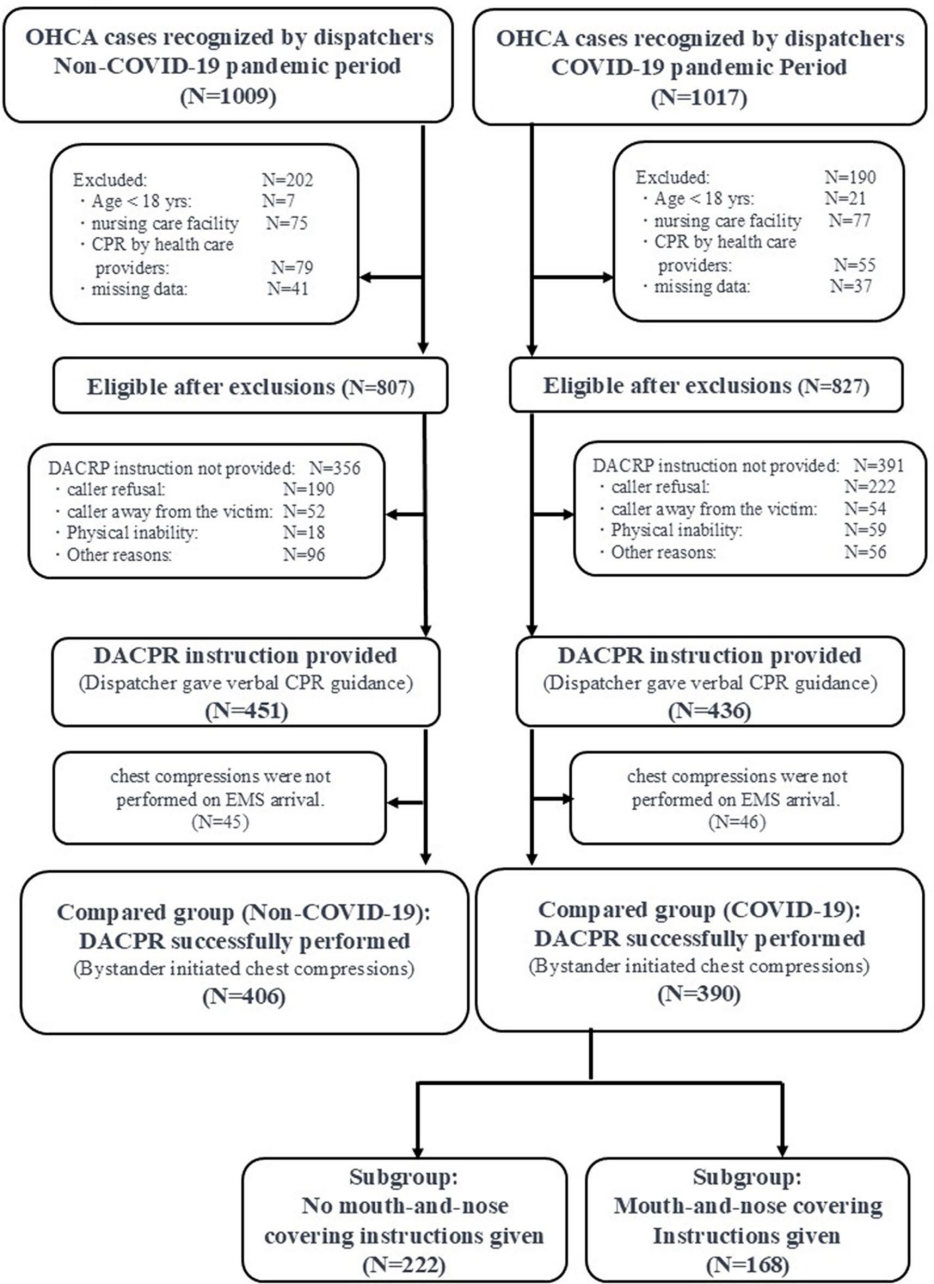
Study flowchart. This flowchart delineates the process of patient inclusion and exclusion for the study, comparing CPR and DACPR during the Coronavirus Disease 2019 (COVID-19) and non-pandemic periods. Abbreviations: CPR, Cardiopulmonary Resuscitation; DACPR, Dispatcher-Assisted Cardiopulmonary Resuscitation; EMS, Emergency Medical Services

Figure 1 presents the inclusion criteria for this study, which included all cases identified as OHCA by dispatchers during the study period. The exclusion criteria were as follows: (1) age < 18 years, (2) cases in nursing care facilities, (3) cases involving healthcare providers before EMS arrival, (4) missing data, (5) absence of DACPR instructions, and (6) no chest compressions performed upon EMS arrival. Eligibility was determined retrospectively by reviewing recorded emergency calls and DACPR logs, following a standardized assessment protocol. Information on whether the cardiac arrest was traumatic or non-traumatic was not available in the DACPR dataset. While dispatchers may initiate CPR instructions in cases where the patient is unresponsive and not breathing regardless of cause, traumatic cases were not explicitly identified in the records and therefore could not be selectively included or excluded.

### Variables and data collection

This study utilized dispatcher audio recordings and EMS field reports from Nara Prefecture to extract variables related to DACPR performance.

Patient demographics included age, sex, and whether the patient was older than 65 years. Arrest characteristics included whether the arrest was witnessed, the location of the event (categorized as home, public place, on the street, workplace, or other), and whether it occurred indoors or outdoors. The bystander’s relationship to the patient was classified as family member, acquaintance, passerby, or other.

Key time intervals in the DACPR process were defined based on the framework established by Dameff et al. [17] and included the following (in seconds):

**T1: **Time from emergency call receipt to recognition of cardiac arrest.

This was recorded at the moment when the caller responded “no” to both questions regarding consciousness and normal breathing, and the dispatcher identified OHCA.

**T2:** Time from emergency call receipt to initiation of CPR instructions.

This corresponded to the point when the dispatcher instructed the caller to place the patient on a firm surface [18, 19] and kneel beside them.

**T3:** Time from emergency call receipt to the start of chest compressions.

This was defined as the moment chest compressions were audibly confirmed during the call (e.g., verbal counting or characteristic compression sounds). Time intervals were measured by dispatchers reviewing the audio recordings. If instructions were not completed, reasons such as caller refusal, disconnection, or inability to perform CPR were documented. CPR performance and infection prevention behaviors were also evaluated. Upon EMS arrival, the quality of bystander CPR was classified as follows:

- **Chest Compression in Progress**: Ongoing compressions observed at the scene.
- **Good Chest Compression**: Appropriate rate, depth, and hand position.
- **Poor Chest Compression**: Inadequate technique, use of a soft surface, or suboptimal posture [20].

During the COVID-19 pandemic, the DACPR protocol was modified to include infection control instructions. After OHCA recognition, dispatchers were instructed to:

1. Ask whether the bystander was wearing a mask,
2. Instruct the bystander to don a mask and cover the patient’s mouth and nose with a cloth or mask,
3. Proceed with chest compression instructions.

Adherence to these infection prevention steps and the mask-wearing status of both bystanders and patients were documented by EMS personnel upon arrival.

The primary outcome was the time from call receipt to the initiation of chest compressions (T3). Secondary outcomes included the intermediate time intervals T1 (time from call receipt to recognition of cardiac arrest); T2 (time from call receipt to initiation of CPR instructions); the proportion of cases where DACPR was initiated; the quality of chest compressions as assessed by EMS personnel; and adherence to infection prevention instructions (masking of patient and/or bystander).

### Study size

All eligible cases of dispatcher-identified OHCA with attempted DACPR during the study periods were included. No a priori sample size calculation was performed.

### Statistical methods

Continuous variables were summarized as medians with interquartile ranges, and categorical variables as numbers with percentages. The normality of continuous variables (T1, T2, and T3) was assessed using the Shapiro-Wilk test. Given that the data were not normally distributed (*p* <.01 for all), non-parametric statistical methods were applied: Mann-Whitney U tests for comparisons of continuous variables and Kruskal–Wallis tests for comparisons among three or more groups. Chi-square tests were used for categorical variables. In addition, subgroup analyses were conducted within DACPR successfully performed cases to examine differences in CPR initiation time (T3) based on the presence or absence of mouth-and-nose covering instructions, and according to the adherence status to these instructions. Mann-Whitney U tests or Kruskal–Wallis tests were used for these comparisons as appropriate. To evaluate whether infection prevention instructions affected the time to chest compression initiation (T3, the primary outcome), we performed a multivariable linear regression analysis including only cases in which DACPR was successfully performed. Explanatory variables included the presence or absence of mouth-and-nose covering instructions, patient age > 65 years, sex, witness status, COVID-19 period, and location of arrest (inside vs. outside the home). Regression coefficients (β) with 95% confidence intervals (CIs) and *P* values were reported.

All statistical analyses were performed with EZR (Saitama Medical Center, Jichi Medical University, Saitama, Japan), which is a graphical user interface for R (The R Foundation for Statistical Computing, Vienna, Austria, version 4.2.2). More precisely, it is a modified version of the R Commander (version 1.61) designed to add statistical functions frequently used in biostatistics [21]. *P* values were considered statistically significant at < 0.05.

## Results

### Study population and characteristics

Dispatchers identified cardiac arrest in 1,009 and 1,017 cases during the non-COVID-19 and COVID-19 pandemic periods, respectively, of which 807 (80.0%) and 827 (81.3%) met the study’s inclusion criteria (Fig. 1). Of these, dispatchers provided the DACPR protocol in 451 cases (55.9%) during the non-COVID-19 pandemic period and in 436 cases (52.7%) during the COVID-19 pandemic period, with no statistical difference between the two groups (*p* =.214). The final study population for analysis of the primary outcome (T3) consisted of 406 cases in the non-pandemic period and 390 cases in the pandemic period, in which bystanders were confirmed to be performing chest compressions upon EMS arrival. The proportion of these cases was similar between the two periods (50.3% vs. 47.2%, *p* =.825). Among the 390 cases during the pandemic period, dispatchers provided mouth-and-nose covering instructions in 168 cases (43.1%), while such instructions were not provided in the remaining 222 cases (56.9%).

Table 1 summarizes the characteristics of cases in which DACPR was successfully performed (Compared Group in Fig. 1) during both periods. There were no statistically significant differences in age, sex, witness status, location of arrest, or bystander relationship to the patient between the non-pandemic and pandemic periods.

**Table 1:** Patient and bystander characteristics

	Non-COVID-19 pandemic period *N* = 406	COVID-19 pandemic period *N* = 390	*P* values
Patient Age, yrs	78.0 (67.0–87.0)	80.0 (70–87)	0.190
Patient age over 65, n (%)	308 (75.9)	317 (81.3)	0.070
Male, n(%)	202 (51.0)	215 (55.1)	0.271
Witnessed arrest, n(%)	83 (20.4)	88 (22.6)	0.626
environmental setting of arrest (outdoor/indoor)	21/385	24/366	0.646
Location of the arrest			
Home, n(%)	372 (91.6)	366 (93.8)	0.375
Public place, n(%)	12 (3.0)	8 (2.0)	
On the street, n(%)	5 (1.2)	6 (1.5)	
Workplace, n(%)	8 (2.0)	2 (0.5)	
Others, n(%)	9 (2.2)	8 (2.1)	
Relationship with patient:			
Family member, n(%)	348 (85.7)	352 (90.3)	0.055
Acquaintance, n(%)	19 (4.7)	20 (5.1)	
Passerby, n(%)	31 (7.6)	14 (3.6)	
Others, n (%)	8 (2.0)	4 (1.0)	
Good chest compression on EMS arrival, n (%)	202 (49.8)	221 (56.7)	0.055

Values are reported either as n (%) or median (interquartile range, IQR)

Abbreviations: COVID-19, Coronavirus Disease 2019; EMS, emergency medical services

### Primary outcome: time to chest compression start (T3)

Table 2 shows the key time intervals related to DACPR. The median time to chest compression initiation (T3) was significantly longer during the COVID-19 period compared to the non-COVID-19 period (246.0 s vs. 261.5 s, *p* <.05).

**Table 2:** DACPR time intervals between the non-COVID-19 and COVID-19 pandemic periods

	Non-COVID-19 pandemic period *N* = 406	COVID-19 pandemic period *N* = 390	*P* values
T1: Time to dispatcher’s recognition of cardiac arrest, (s)	71.0 (49.00–114.75)	66.5 (45.0–121.50)	0.379
T2: Time to CPR instruction start, (s)	188.5 (143.00–249.75)	196.5 (135.25–273.50)	0.613
T3: Time to chest compression start, (s)	246.0 (186.25–314.75)	261.5 (190.0–347.75)	< 0.05*

Data are presented as median (interquartile range, IQR)

All time intervals (T1–T3) are measured from the time of emergency call receipt

Abbreviations: CPR, Cardiopulmonary Resuscitation; DACPR, Dispatcher-Assisted Cardiopulmonary Resuscitation; COVID-19, Coronavirus Disease 2019

### Secondary outcomes

The frequencies of “Good chest compression” verified by EMS on arrival were also similar between the two groups (202 cases; 49.8% vs. 221 cases; 56.7%) (Table 1). The median values for T1 and T2 did not significantly differ between groups (T1: 71.0 s vs. 66.5 s, *p* =.379; T2: 188.5 s vs. 196.5 s, *p* =.349; Table 2).

Table 3 compares DACPR cases with and without mouth-and-nose covering instructions. The rate of bystanders wearing masks was significantly higher when instructions were provided (107 cases, 64.8% vs. 13 cases, 5.9%; *p* <.01). Similarly, the proportion of patients with their mouth and nose covered was significantly higher when such instructions were given (42 cases, 25.6% vs. 2 cases, 0.9%; *p* <.01). Among cases during the pandemic, the T2 and T3 intervals were nearly identical between those with and without covering instructions.

**Table 3:** DACPR time intervals with and without mouth-and-nose covering instructions

	No instructions (*n* = 222)	Mouth-and-nose covering instructions (*n* = 168)	*P* value
Proportion of bystanders wearing masks (%)	13 (5.9)	107 (64.8)	< 0.01
Proportion of patients with mouth-and-nose covered (%)	2 (0.9)	42 (25.6)	< 0.01
T1: Time to dispatcher’s recognition of cardiac arrest, (s)	73.0 (47.25–130.0)	60.0 (40.0–102.25)	< 0.05*
T2: Time to CPR instruction start, (s)	194.0 (137.75–274.75)	197.0 (135.75–266.25)	0.718
T3: Time to chest compression start, (s)	260.5 (182.0–354.0)	263.5 (195.75–341.25)	0.741

Values are reported either as n (%) or median (interquartile range, IQR)

All time intervals (T1–T3) are measured from the time of emergency call receipt

Abbreviations: CPR, Cardiopulmonary Resuscitation;　DACPR, Dispatcher-Assisted Cardiopulmonary Resuscitation

Table 4 shows a detailed analysis of mouth-and-nose covering instruction adherence, categorized into four groups based on the actual mask-wearing status of bystanders and patients upon EMS arrival. The largest group was Group 2, in which only bystanders wore masks (*N* = 71, 42.2%), followed by Group 4, in which neither bystanders nor patients wore masks (*N* = 55, 32.7%). The median T3 time intervals were almost identical between these two groups (261.0 vs. 260.0 s). Group 1, where both bystanders and patients were masked (*N* = 36, 21.4%), had the longest median T3 interval (277.0 s). However, the differences across the four groups were not statistically significant.

**Table 4:** DACPR time intervals based on adherence to the mouth-and-nose covering instruction

	Group 1: Both masked (*N* = 36)	Group 2: Bystander only (*N* = 71)	Group 3: Patients only (*N* = 6)	Group 4: Neither masked (*N* = 55)	*P* values
Patient mask	Yes	No	Yes	No	
Bystander mask	Yes	Yes	No	No	
T1: Time to dispatcher’s recognition of cardiac arrest, (s)	60.0 (39.75–101.0)	69.0 (44.0–104.0)	62.5 (57.5–162.75)	57.0 (39.0–95.0)	0.712
T2: Time to CPR instruction start, (s)	185.5 (134.0–237.5)	200.0 (137.5–273.0)	174.0 (125.0–232.0)	210.0 (144.0–281.0)	0.650
T3: Time to chest compression start, (s)	277.0 (207.75–336.0)	261.0 (184.0–335.5)	245.0 (193.5–302.5)	260.0 (198.5–365.5)	0.681

Data are presented as median (interquartile range, IQR)

All time intervals (T1–T3) are measured from the time of emergency call receipt

Abbreviations: CPR, Cardiopulmonary Resuscitation; DACPR, Dispatcher-Assisted Cardiopulmonary Resuscitation

Table 5 presents the results of the multivariable regression analysis assessing the impact of the COVID-19 pandemic period on T3. The pandemic period itself was not significantly associated with T3 (*β* = 14.597, *p* =.072). Other variables, including patients aged over 65 (*β* = 17.625, *p* =.078) and location of cardiac arrest (*β* = -23.128, *p* =.196), were also not significantly associated with T3 prolongation. The multiple R-squared of the model was 0.0108, and the adjusted R-squared was 0.0046, indicating that the overall explanatory power of the model was limited.

**Table 5:** Multivariable linear regression analysis of factors associated with T3

Variable	T3: Time to chest compression start, (s)
Patient age over 65 (Yes = 1, No = 0)	17.625 (-1.986, 37.236) *p* =.078
Sex (female = 1, male = 0)	-4.334 (-20.504, 11.736) *p* =.600
Witness (Yes = 1, No = 0)	-2.801 (-21.947, 16.345) *p* =.774
COVID-19 Period (Yes = 1, No = 0)	14.597 (-1.330, 30.524) *p* =.072
Location of cardiac arrest (outside the home = 0, at home = 1)	-23.128 (-58.241, 11.986) *p* =.196

Data are presented as regression coefficient (β), 95% Confidence Interval (CI), and p-value

T3 is measured from the time of emergency call receipt

## Discussion

In this study, the proportion of DACPR was not substantially altered after the implementation of the modified DACPR protocol during the COVID-19 period in Nara, Japan (55.9% vs. 52.7%, *p* =.214). However, the introduction of the modified DACPR protocol, which included instructions for covering the mouth-and-nose before starting chest compressions, resulted in a statistically significant delay of approximately 15.0 s in DACPR initiation (T3: 246.0 s vs. 261.5 s, *p* <.05). Considering the relatively low adherence to the modified protocol, this observed delay might underestimate the true impact in scenarios with higher compliance. Only 21.4% of cases (36/168) with mask-related instructions showed full adherence, defined as both the bystander and the patient covering their mouth and nose. Among these fully compliant cases (Group 1), the median time to DACPR initiation (T3) was 277.0 s, showing a 17.0-s delay compared to cases with no mask use (Group 4: 260.0 s), suggesting that full adherence to infection prevention measures may delay DACPR initiation. Additionally, the study was conducted across a geographically broad and demographically diverse region, encompassing urban and rural areas. With dispatchers identifying approximately 1,000 OHCA cases annually during the study period, the sample was robust and varied. These features support the generalizability of our findings to similar settings.

We believe that the observed delay in T3 during the COVID-19 pandemic period may have been driven by factors beyond bystander composition, specifically the high prevalence of family-member bystanders combined with pandemic-specific psychosocial influences. In our study, although the proportions of home-based OHCAs and family-member bystanders remained consistently high across both periods (home: 91.6% vs. 93.8%, *p* =.375; family member: 85.7% vs. 90.3%, *p* =.055), we observed a significant delay in T3 during the pandemic period. Some studies have suggested that family-member bystanders may be more hesitant to perform CPR compared to other groups. For example, Tuffley et al. reported that emotionally distressed callers faced barriers to performing CPR and required additional encouragement from dispatchers [22]. Similarly, Tanaka et al. found that family-member bystanders were less likely to initiate CPR and tended to experience delays in calling EMS and starting chest compressions [23]. These tendencies may have been further amplified during the pandemic, when fear of infection and psychological stress were heightened. Supporting this hypothesis, several studies during the COVID-19 pandemic reported a decrease in bystander CPR rates, attributed to the rise in home-based OHCAs and infection-related concerns [9–11]. These pandemic-specific factors may have intensified hesitation among family-member bystanders, contributing to delays in CPR initiation despite dispatcher assistance. However, contrasting findings have emerged; for example, a study in Singapore [24] found that BCPR rates remained stable, while a study in London [25] observed an increase. These variations may partly reflect regional differences in CPR education, dispatcher protocols, and EMS systems, suggesting that social and cultural factors could also influence bystander responses during a pandemic.

During the COVID-19 pandemic, dispatcher adherence to the infection prevention component of the modified DACPR protocol remained relatively low (43.1%). Several challenges hindered consistent implementation. In-house interviews with dispatchers revealed that some prioritized the rapid initiation of CPR and unintentionally omitted infection prevention guidance, while others reported that distressed or panicked callers made it difficult to convey additional instructions. These findings suggest that, in high-stress situations, dispatchers may prioritize life-saving actions over infection control, limiting adherence to pandemic-specific protocols. This interpretation is supported by a recent study from Austria, which found that emergency control center dispatchers experienced greater psychological stress and poorer recovery than frontline EMS personnel during the early waves of the COVID-19 pandemic, likely due to the mental burden of remotely managing emergencies under time pressure and emotional strain [26].

As shown in Table 4, Group 1—where both the patient and the bystander wore masks or equivalent coverings—exhibited the longest T3 time (277.0 s), suggesting that full adherence to infection prevention instructions may significantly delay DACPR initiation. Group 2—where only the bystander wore a mask—was the most common pattern (41.7%) and showed a T3 time (261.0 s), nearly identical to that of Group 4 (260.0 s), where neither party was masked. These patterns suggest that partial adherence to infection prevention instructions may have a limited effect on CPR initiation time compared to full adherence. Interestingly, Group 3—where only the patient’s mouth and nose were covered—had a shorter T3 time (247.5 s) than Group 4, though this counter-intuitive finding is likely attributable to random variation due to the small sample size (*n* = 6) and should be interpreted with caution. Overall, while complete adherence to infection prevention measures appears to be associated with delayed DACPR initiation, such cases were relatively rare in our dataset.

Although the COVID-19 period itself was not identified as a statistically significant predictor of delays in DACPR initiation in our multivariable analysis (*p* =.072), the low adherence rate to infection prevention instructions during the pandemic may have underestimated its true impact on T3. Additionally, several other factors may have contributed to the observed delays. For example, patient repositioning challenges in home environments—such as limited space, soft surfaces, or obstacles—have been reported as barriers to timely CPR [27, 28]. In our analysis, “location” was simply categorized as home or outside the home, which may not have fully captured such complexities. While age over 65 was also not statistically significant in our analysis (*p* =.078), potential barriers such as limited physical strength among elderly households may contribute to CPR delays and warrant further investigation. These findings highlight the need for future research to explore more detailed environmental and behavioral factors. Future emergency response protocols for infectious disease outbreaks should aim to minimize delays while ensuring necessary infection prevention measures.

Finally, although our primary focus was on the impact of infection prevention instructions on DACPR performance, it is important to acknowledge that the median T3 time in our setting exceeded the American Heart Association’s recommended benchmark of 150 s from call receipt to first chest compression [29]. One potential contributing factor is the operational structure of Japan’s emergency dispatch system, where telecommunicators are part of the fire department and typically rotate every 2 years, which may limit the accumulation of specialized experience. Continued efforts to streamline protocols and strengthen dispatcher training are warranted, and there is room for improvement in this area.

### Limitations

This study has several limitations. First, this study was conducted in Nara, a prefecture in Japan. While our findings may be applicable to other regions with similar demographic and emergency medical service characteristics, it remains uncertain whether the same results would be observed in areas with substantially different EMS systems, cultural contexts, or bystander behaviors.

Second, most bystanders in both pandemic and non-pandemic periods were family members. Although family members may be more willing to initiate CPR in familiar environments, prior studies have reported that they are also more prone to delays in calling EMS and initiating chest compressions due to emotional stress or uncertainty [23]. We did not evaluate these psychosocial aspects in our study.

Third, some bystanders or patients might have been wearing face masks before the cardiac arrest event, independent of dispatcher instructions. Although most OHCAs in our study occurred at home—where mask use is likely less common—we could not confirm the exact number of such cases. This limitation introduces some uncertainty regarding the extent to which the modified DACPR protocol alone influenced the T3 interval.

Fourth, we were unable to collect data regarding the impact of COVID-19 morbidity among patients and bystanders, as no systematic follow-up data were available. While the fire department did not receive any claims of infection transmission following CPR events, this remains an area for further investigation. Additionally, our study did not include patient outcome data such as return of spontaneous circulation or information on the receiving hospital, as the DACPR records used in this study did not contain follow-up clinical or hospital transport destination data. Although linkage with the National Utstein-style databases could theoretically allow for outcome assessment, such integration may result in a reduced sample size due to incomplete matching. While these outcomes are clinically important, the primary aim of this study was to evaluate the performance and timing of DACPR rather than clinical prognosis. Fifth, because data were collected retrospectively from dispatcher audio recordings and EMS field reports, there is potential for information bias, including inaccuracies in call transcription or subjective assessment of CPR quality. However, the use of dispatcher-reviewed audio recordings for measuring time intervals may have improved the reliability of these measurements.　Finally, the explanatory power of our multivariable regression model was limited. This suggests that unmeasured or unquantified behavioral and situational factors may have influenced CPR initiation time, and future studies should incorporate additional variables to improve model fit.

## Conclusions

In this study, the proportion of DACPR provided by dispatchers and the rate of bystander-performed chest compressions upon EMS arrival were comparable between the pre-pandemic and pandemic periods. However, CPR initiation (T3) was modestly but significantly delayed during the pandemic period. Although multivariable analysis did not identify the pandemic period itself as a statistically significant predictor of this delay, this may be partly explained by the low adherence rate to the infection prevention instructions during the pandemic. Further research is needed to explore additional environmental and behavioral factors influencing DACPR performance and to refine protocols for future emergency response situations.

## Data Availability

No datasets were generated or analysed during the current study.

## Abbreviations

CPR: Cardiopulmonary resuscitation
DACPR: Dispatcher-Assisted Cardiopulmonary Resuscitation
BCPR: Bystander cardiopulmonary resuscitation
OHCA: Out-of-Hospital Cardiac Arrest
EMS: Emergency Medical Service
